# Extracorporeal Carbon Dioxide Removal: From Pathophysiology to Clinical Applications; Focus on Combined Continuous Renal Replacement Therapy

**DOI:** 10.3390/biomedicines11010142

**Published:** 2023-01-05

**Authors:** Francesca Cappadona, Elisa Costa, Laura Mallia, Filippo Sangregorio, Lorenzo Nescis, Valentina Zanetti, Elisa Russo, Stefania Bianzina, Francesca Viazzi, Pasquale Esposito

**Affiliations:** 1 Unit of Nephrology, Dialysis and Transplantation, Ospedale Policlinico San Martino, 16132 Genova, Italy; 2 Department of Internal and Medical Specialities (DIMI), University of Genoa, 16132 Genova, Italy; 3 Unit of Nephrology, Ospedale San Luca, 55100 Lucca, Italy; 4 Neonatal and Pediatric Intensive Care Unit, Emergency Department, IRCCS Istituto Giannina Gaslini, 16146 Genova, Italy

**Keywords:** extracorporeal CO_2_ removal, hypercapnia, acute respiratory distress syndrome, lung-protective ventilation, continuous renal replacement therapy, Coronavirus disease 2019, acute kidney injury, lung–kidney crosstalk

## Abstract

Lung-protective ventilation (LPV) with low tidal volumes can significantly increase the survival of patients with acute respiratory distress syndrome (ARDS) by limiting ventilator-induced lung injuries. However, one of the main concerns regarding the use of LPV is the risk of developing hypercapnia and respiratory acidosis, which may limit the clinical application of this strategy. This is the reason why different extracorporeal CO_2_ removal (ECCO_2_R) techniques and devices have been developed. They include low-flow or high-flow systems that may be performed with dedicated platforms or, alternatively, combined with continuous renal replacement therapy (CRRT). ECCO_2_R has demonstrated effectiveness in controlling PaCO_2_ levels, thus allowing LPV in patients with ARDS from different causes, including those affected by Coronavirus disease 2019 (COVID-19). Similarly, the suitability and safety of combined ECCO_2_R and CRRT (ECCO_2_R–CRRT), which provides CO_2_ removal and kidney support simultaneously, have been reported in both retrospective and prospective studies. However, due to the complexity of ARDS patients and the limitations of current evidence, the actual impact of ECCO_2_R on patient outcome still remains to be defined. In this review, we discuss the main principles of ECCO_2_R and its clinical application in ARDS patients, in particular looking at clinical experiences of combined ECCO_2_R–CRRT treatments.

## 1. Introduction

Respiratory failure is defined as a failure of the lung to oxygenate the arterial blood adequately and/or to prevent carbon dioxide (CO_2_) retention [1]. Different types of respiratory failure are associated with various degrees of hypoxemia and CO_2_ retention; hypercapnia usually is associated with hypoventilation and ventilation–perfusion inequality. Most patients with respiratory failure require mechanical ventilation (MV), and in some cases, extracorporeal respiratory support as well [2]. These therapies encompass extracorporeal membrane oxygenation (ECMO) and the extracorporeal CO_2_ removal system (ECCO_2_R). ECMO takes over the gas exchange function of the lungs, ensuring full oxygenation and CO_2_ removal. ECCO_2_R is a CO_2_ removal system that does not affect oxygenation, whose principal aim is enabling lung-protective MV (LPV) by limiting the risks of ventilator-induced lung injuries (VILIs) [3]. In this review, we discuss the principles of ECCO_2_R and its main clinical applications, focusing on experiences of the use of ECCO_2_R in combination with continuous renal replacement therapy (CRRT) in patients with or without renal failure.

## 2. Carbon Dioxide and Acid–Base Balance

CO_2_ is produced in mitochondria as the ‘end product’ of aerobic metabolism, and it is carried in the blood in different forms. The normal arterial partial pressure of CO_2_ (PaCO_2_) is 37 to 43 mmHg. A part of CO_2_ is dissolved in the blood (about 5%), and it is the fraction available for removal with an extracorporeal system. Other ways of CO_2_ carriage are through bicarbonate (HCO_3_^−^) and carbamino compounds [4]. Carbamino compounds, comprising about 20% of the total CO_2_, are formed by the combination of CO_2_ with terminal amine groups of blood proteins, of which the most important is the globin of hemoglobin (carbaminohemoglobin). Bicarbonate is the principal storage of CO_2_ (about 70%), and it is formed by the following reaction:

CO_2_ + H_2_O ← → H_2_CO_3_ ← → HCO_3_^−^ + H^+^

The combination of CO_2_ with free water (H_2_O) to form carbonic acid (H_2_CO_3_) is catalyzed in red blood cells and on pulmonary capillaries’ membranes by carbonic anhydrase, which is not present in plasma. At physiologic pH ranges, 96% of carbonic acid is dissociated in HCO_3_^−^ and hydrogen ion (H^+^) [5]. The reverse reaction, which generates CO_2_ from HCO_3_^−^, follows linear kinetics and does not saturate; therefore, CO_2_ diffuses more efficiently than O_2_ and is almost not affected by the hemoglobin concentration [2,3]. HCO_3_^−^ and CO_2_ are the main components driving pH and follow the formula:

pH = 6.1 + log [HCO_3_^−^]/[CO_2_] = 6.1 + log [HCO_3_^−^]/0.03 × [PaCO_2_]

The lungs eliminate over 10.000 mEq of carbon acid every day, and they are the main system that compensates for the metabolic alteration of the acid–base status. Respiratory acidosis often develops in cases of hypercapnic respiratory failure, driven by the augmentation of CO_2_ and the reduction in the HCO_3_^−^/CO_2_ ratio. This alteration in the pH is even more important in the case of both acute kidney injuries (AKIs) and chronic kidney damage (CKD) because the capacity of the kidney for HCO_3_^−^ reabsorption is blunted or ineffective.

## 3. ECCO_2_R: Principles 

### 3.1. Principles and Systems

A working ECCO_2_R system requires vascular access, a blood pump, a membrane lung, an exchange gas, and anticoagulation [6]. ECCO_2_R devices have two different configurations: venovenous (VV-ECCO_2_R) and artero-venous (AV-ECCO_2_R) [7,8]. AV-ECCO_2_R is performed via arterial and venous cannulation, usually femoral, with 15 French cannulas, using the arterial blood pressure to pump the blood inside the circuit. So, the blood flow depends exclusively on the cardiac output of the patient [9]. This technique is invasive, less effective in hypotension, and can have many complications, so it is not widely used. In VV-ECCO_2_R, blood is drawn from a central vein by a draining cannula using centrifugal, roller, non-occlusive, or diagonal flow magnetic rotary pumps, which generates a pressure gradient and permits a flow across the circuit. This approach allows ECCO_2_R by utilizing small central venous catheters, commonly introduced via the right internal jugular vein [10]. The core of the ECCO_2_R circuit is the gas exchange membrane, a device with a complex geometry based on hollow fibers. The membrane material is poly-4-methyl-1-pentene (PMP), which represents the most used configuration because it reduces plasma leakage and permits gas transfer by diffusion, avoiding direct blood–gas contact. The exchange surfaces of the membranes differ in size from 0.32 to 0.65 m^2^ for low-flow VV systems and 1.3 m^2^ for high-flow VV and AV systems. Circuits and membranes are coated with heparin to improve biocompatibility and gas exchange, as well as too limit capillary leakage. The extraction of carbon dioxide is performed through the sweeping of the membrane by a fresh gas (O_2_ or medical air) devoid of CO_2_ [1,2,3,4,5]. The main determinants of CO_2_ removal in ECCO_2_R are extracorporeal blood flow, the PaCO_2_ gradient, sweep gas flow, and membrane size and characteristics [11]. According to the blood flow rate, we can distinguish between low-flow VV-ECCO_2_R systems operating with a blood flow rate between 200 and 400 mL/min, and high-flow systems (i.e., blood flow rate higher than 500 mL/min). The potential advantages of low-flow systems include the possibility of using conventional CRRT platforms and dual-lumen dialysis catheters, whereas high-flow systems require dedicated devices. Regarding the CO_2_ removal efficiency, in theory, an augmentation of blood flow should result in a linear increase in CO_2_ removal. So, considering that 1 L of blood transports around 500 mL of CO_2_, and that an average adult produces 250 mL/min of CO_2_, a blood flow rate of 200–300 mL/min may permit the removal of about 50% of the total CO_2_ produced, while an increase in the blood flow rate > 500 mL/min may remove all the produced CO_2_. However, experimental evidence suggests that, due to the limitations of blood flow and membrane efficiency, the actual removal capacity is inferior and, in particular, low-flow systems may remove up to 25% of the carbon dioxide produced [12]. Blood flow is only one of the determinants of CO_2_ removal [13]. Indeed, the CO_2_ transfer follows a diffusion gradient according to Fick’s law, so the difference between the blood flow and sweep gas in terms of CO_2_ pressure has a crucial role. In the sweep gas, the PaCO_2_ tends to be as low as possible (or even absent). Then, the venous blood partial pressure sustains the diffusion gradient. As the CO_2_ diffuses and achieves equilibrium almost instantaneously, the sweep gas flow rate is crucial to keeping the CO_2_ low on the gas side of the membrane [14]. The CO_2_ removal has a linear relationship with the sweep gas flow until a threshold of 4–5 L/min; after that there is no augmentation of the CO_2_ removal [15]. In addition, the membrane surface also has a relevant impact on the CO_2_ diffusion, and it is proportional to the quantity of the gas exchange and CO_2_ removal. Furthermore, large membranes carry a higher thrombotic risk, while small ones have an increased risk of haemolysis. Interestingly, as an innovative approach, there are some experimental studies on the application of hollow fibers coated with immobilized carbonic anhydrase to enhance the conversion of carbonic acid to CO_2_ [16].

### 3.2. Anticoagulation

As for other extracorporeal circuits, anticoagulation is required to prevent thromboembolic complications, especially for low-flux ECCO_2_R systems that are at a high risk of circuit clotting. No standard anticoagulation strategy for ECCO_2_R has been established yet. Among the different options, the most used in clinical trials and daily practice is systemic anticoagulation with heparin, which may be provided with unfractionated heparin or low-molecular-weight heparin. The major adverse effects of this strategy are bleeding and heparin-induced thrombocytopenia [17]. A promising alternative is citrate-based regional anticoagulation. Trisodium citrate infused at the beginning of the extracorporeal circuit binds to calcium, inhibiting the activation of calcium-dependent coagulation factors. The infusion of calcium chlorate at the circuit end reverses the citrate effect before the blood returns to the patient [18]. This strategy may reduce the incidence of hemorrhagic complications and can also improve the ECCO_2_R circuit survival time [19]. Moreover, the administration of trisodium citrate leads to the formation of sodium bicarbonate, an end-product of citrate metabolism, which might buffer the excess acid [20].

### 3.3. Complications

There are many complications related to the use of ECCO_2_R. They can be divided into three groups: patient-related, catheter-related, and device-related [10]. The most frequent adverse event is the occurrence of bleeding events (cerebral, gastrointestinal, and nasopharyngeal), mainly caused by the necessity of systemic anticoagulation [21]. Other commonly observed complications are thrombocytopenia and hemolysis. Distal limb ischemia and compartment syndrome of the lower limb (requiring fasciotomy or limb amputation) are associated with arterial cannulation [22]. Otherwise, venous catheterization can present more common complications, such as catheter-site bleeding, malposition, and infection. Vascular thrombosis occurs more often during low-flow VV-ECCO_2_R because of the increased exposure time to the membrane lung and circuit. Finally, device alterations can lead to pump or oxygenator failure, heat exchanger malfunction, or clot formation [21]. 

## 4. ECCO_2_R: Clinical Applications 

While ECMO can ensure blood oxygenation and decarboxylation, ECCO_2_R provides partial respiratory support by removing CO_2_ with minimal impact on blood oxygenation [23]. The optimization of CO_2_ removal may allow for a proper ventilatory strategy in patients with respiratory failure. Indeed, these patients often require invasive MV (IMV), which may present some harmful effects, such as VILI, especially in cases in which a high tidal volume (TV) and plateau pressure (Pplat) are used [24]. The most recognized strategy to avoid VILI is lung-protective ventilation (LPV), which has the advantage of a low TV and Pplat. The introduction of these strategies in clinical practice has constituted a significant advance in the care of patients with respiratory failure [25]. However, one of the main concerns regarding the use of LPV is the risk of developing hypercapnia and respiratory acidosis, which are independently associated with increased adverse effects, including increased mortality [26]. In particular, hypercapnia may increase intracranial pressure and exert vasoconstrictive effects on pulmonary circulation, leading to pulmonary hypertension and augmented right ventricular afterload [9]. These are why ECCO_2_R techniques and devices have been developed, thus allowing LPV in cases of respiratory failure [27]. Moreover, ECCO_2_R may also be used to sustain the reduction in ventilation pressures in cases of non-intubated patients, thus preventing the demand for intubation. Given these objectives, the treatment of hypercapnic respiratory acidosis consequent to chronic obstructive pulmonary disease (COPD) and acute respiratory distress syndrome (ARDS) constitutes the main clinical indication for ECCO_2_R.

### 4.1. ECCO_2_R in COPD

COPD represents a condition of chronic hypercapnia that may worsen during acute exacerbations (ae-COPD). In this case, hypercapnia may be generated because of the reduced CO_2_ removal due to alveolar overdistension and the ventilation/perfusion imbalance, as well as increased CO_2_ production secondary to respiratory muscle work [28]. Although non-invasive ventilation (NIV) represents the first-choice treatment for ae-COPD [29], NIV failures often occur, and endotracheal intubation and IMV may be required [23]. ECCO_2_R therapy is an emerging option for managing hypercapnia while allowing LPV in these cases [27]. The use of ECCO_2_R in patients with ae-COPD enhances CO_2_ removal, lowers the respiratory rate, prolongs the expiratory time, and minimizes positive end-expiratory pressure (PEEP) [9]. Moreover, there is a reduction in the use of respiratory muscles with a consequent decrease in CO_2_ production [23]. Thus, ECCO_2_R devices can reduce NIV failure, preventing the need for IMV, or can facilitate weaning from MV [30,31]. However, it should be recognized that there is no evidence of survival benefits; additionally, ECCO_2_R does not seem to be risk-free in this setting. In a case–control ECLAIR study involving twenty-five COPD patients with acute hypercapnic respiratory failure refractory to NIV, the initiation of the VV-ECCO_2_R treatment was associated with a PaCO_2_ reduction of 17.5 mm Hg at 1 h and 29.5 mm Hg at 24 h accompanied by a 56% reduction in the intubation rate and a 60% reduction in the time on IMV. However, there were no significant effects on the length of patients’ ICU stay and mortality rates; moreover, the treatment was complicated by major adverse events in 11 patients (44%), including 9 patients (36%) with bleeding events [32]. In 2020, a consensus proposed the principal criteria for starting ECCO_2_R in patients with ae-COPD (no decrease in PaCO_2_ and no decrease in respiratory rate while on NIV), as well as patients recently initiated on mechanical ventilation after NIV failure to allow for early extubating. Accordingly, treatment targets for ae-COPD patients receiving ECCO_2_R therapy include comfortable patients, a pH of >7.30/7.35, a respiratory rate of <20–25 breaths/min, a decrease in PaCO_2_ by 10/20%, weaning from NIV, a decrease in HCO_3_^−^, and the maintenance of hemodynamic stability [33].

### 4.2. ECCO_2_R in ARDS

ARDS is a life-threatening syndrome in which the respiratory system fails in the gas exchange function of oxygenation and/or carbon dioxide elimination. The mortality rate from ARDS is approximately 40 to 50%, and IMV is required in almost all patients [34,35]. However, in some patients, hypoxia and/or hypercapnia are refractory to MV despite maximal tolerable ventilation settings. A landmark trial by the ARDSNet group demonstrated that ventilating ARDS patients with an LPV modality with a low TV of 6 mL/kg for their predicted body weight (PBW) compared with a traditional TV of 12 mL/kg PBW significantly decreased mortality [36]. However, subsequent results showed lung hyperinflation still occurs in approximately 30% of ARDS patients ventilated with the ARDSNet strategy [37,38]. Therefore, the reduction in the TV to 3–4 mL/kg PBW and the Pplat to ≤25 cmH_2_O, otherwise known as ultraprotective ventilation (uLVP), has been proposed to further minimize the risk of VILI [39]. This strategy entails a significant risk of severe hypercapnic respiratory acidosis [40], a condition independently associated with worse outcomes [41,42]. So, the development of hypercapnia may constitute a limitation for the use of LPV and provide the reason why a validated ECCO_2_R method may help provide proper ventilation in ARDS patients. In the SUPERNOVA study, a prospective multicenter study, Combes et al. have shown that ECCO_2_R can minimize respiratory acidosis while applying a uLVP strategy in patients with moderate ARDS (PaO_2_/FiO_2_ 100–200 mmHg, with PEEP ≥ 5 cmH_2_O) [43]. In this study, ninety-five patients were treated with the Hemolung Respiratory Assist System (ALung Technologies, Pittsburgh, PA, USA), the iLA active (Novalung, Heilbronn, Germany), and the Cardiohelp^®^ HLS 5.0 (Getinge Cardiopulmonary Care, Rastatt, Germany) devices. The primary outcome was the number of patients who successfully achieved a TV of 4 mL/kg PBW with their PaCO_2_ not increasing more than 20% from the baseline with the value of the arterial pH > 7.30. Secondary endpoints included the assessment of physiological variables and ECCO_2_R settings as well as the frequency of adverse events. The proportions of patients who achieved ultra-protective settings by 8 h and 24 h were 78% and 82%, respectively. The TV, respiratory rate, minute ventilation, and Pplat were significantly lower at 8 h and 24 h compared to the baseline (*p* = 0.001). Moreover, the PaCO_2_ and PaO_2_/FiO_2_ ratio remained stable, while the pH significantly increased at 8 h (*p* < 0.001). ECCO_2_R was maintained for 5 (range 3–8) days. During the ECCO_2_R treatment, adverse events were reported in 39% of the patients, including two severe adverse events directly attributed to ECCO_2_R (brain hemorrhage and pneumothorax). Overall, 69 patients (73%) were alive on day 28, while fifty-nine patients (62%) were alive at hospital discharge. In conclusion, the authors stated that, despite the effectiveness of ECCO_2_R, the relatively high number of adverse events may call into question the risk/benefit balance of this approach, which should be confirmed in randomized clinical trials. The necessity for more robust evidence has been recently highlighted by the results of the REST trial, a multicenter, randomized, open-label, pragmatic clinical trial, which enrolled 412 adult patients receiving mechanical ventilation for acute hypoxemic respiratory failure [44]. The participants were randomized to receive lower TV ventilation facilitated by ECCO_2_R for at least 48 h (n = 202) or standard care with conventional low-TV ventilation (n = 210). The primary outcome was the all-cause mortality 90 days after randomization. Among the patients with acute hypoxemic respiratory failure, ECCO_2_R associated with a low TV did not significantly reduce the 90-day mortality when compared with the standard low-TV ventilation. However, due to the early termination, the study may have been underpowered to detect clinically relevant differences (the initial target enrolment was 1120 patients). Overall, these data highlight that beyond the strong rationale for using ECCO_2_R in ARDS patients, the available evidence is inconclusive, and there is space for expanding the research on this issue. Finally, it should be mentioned that ECCO_2_R has been used also in patients affected by Coronavirus disease-19 (COVID-19). Akkanti et al. described a cohort of 29 mechanically ventilated patients with ARDS secondary to COVID-19 complicated by severe hypercapnia and respiratory acidosis. In this cohort, ECCO_2_R treatments with the Hemolung Respiratory Assist System (ALung Technologies, Pittsburgh, PA, USA) were associated with an improvement in the acid–base parameters while providing LVP. No treatment-related adverse effects were reported, but the prognosis of these patients remained severe, with an overall survival of 38% [45]. 

## 5. Respiratory and Renal Failure: A Dangerous Interconnection

In critically ill patients, pulmonary and renal damage are often associated, providing evidence of lung–kidney crosstalk [46]. It has been estimated that ventilated patients have a three-fold increase in the risk of AKI; up to 30% of patients with ARDS may present kidney damage to some extent [47]. The mechanisms of lung–kidney interactions are bidirectional and multifaceted. First, during ARDS, renal function may be impaired by hemodynamic alterations, driven by venous congestion, neurohormonal activation, and ischemic injury [48]. It has been proved that MV directly impacts renal perfusion, while blood gas disturbances, and, in particular, hypercapnia, may act as a direct renal vasoconstrictor. In addition, toxic factors, oxidative stress, and MV-induced systemic inflammation may promote renal damage [49]. On the other side of the coin, kidney injury can aggravate pulmonary damage through different mechanisms [50]. Fluid overload and metabolic acidosis can increase respiratory work by inducing alveolar flooding and impairing pulmonary gas exchange. The systemic release of mediators expands pulmonary vascular permeability, lung inflammation, and apoptosis. Finally, the downregulation of the transepithelial electrolyte and water transport leads to respiratory failure [5]. Lung–kidney crosstalk has relevant clinical and therapeutical implications. The combination of AKI and ARDS aggravates the mortality rate by as high as 80% [51]. Furthermore, about 35% to 60% of patients with respiratory failure also need renal replacement therapies (RRTs) [52]. This observation underlines the potential clinical utility of providing simultaneous multiple extracorporeal supports, which may also include the combination of ECCO_2_R circuits with the CRRT platform. The most rational indication for ECCO_2_R coupled with CRRT is the association of hypercapnic respiratory acidosis with renal damage requiring CRRT [53]. However, in practice, the ECCO_2_R–CRRT combination has been also used in patients without renal failure, aiming to provide ECCO_2_R by the standard CRRT system, thus reducing the cost and the complexity of the ECCO_2_R treatment [54].

## 6. Experiences with ECCO_2_R Integrated into CRRT Platforms

**Figure 1:**
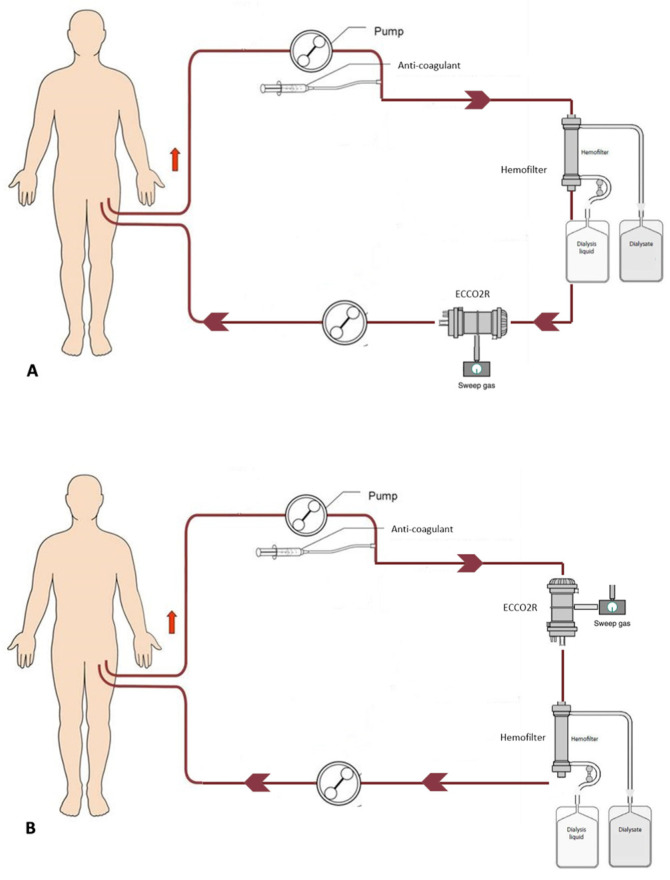
Exemplificative schemes of combined ECCO_2_R–CRRT configurations. In the example CRRT is provided according to CVVHD modality. Membrane oxygenator for ECCO_2_R may be inserted either downstream (**A**) or upstream (**B**) of the hemofilter.

The advantages of integrating a hollow-fiber gas exchanger in a CRRT platform include its simplicity and its potential applicability in non-specialized centers, such as the fact that no additional venous catheter placements are needed. Interestingly, even though ECCO_2_R combined with CRRT had already been under investigation years before the current Coronavirus disease-19 (COVID-19) pandemic, this approach began to attract even more attention during the pandemic due to its potential utility in improving resource allocation. In a pivotal paper in 2009, Terragni et al. tested the possibility of integrating a membrane lung in a modified renal replacement circuit [55]. They used a neonatal membrane with a total membrane surface of 0.33 m^2^ set in a series with a hemofilter to facilitate uLVP in 32 ARDS patients without AKI. They found that the extracorporeal treatment normalized their PaCO_2_ and pH and allowed the use of VT < 6 mL/kg for 144 (84–168) h, which in turn was associated with an improvement in the lung structure and a reduction in the pulmonary cytokines concentration. Following these results, in 2013, Forster et al. reported their experience with a low-flow hollow-fiber gas exchanger implemented in a CRRT circuit in 10 patients with combined respiratory and renal failure [56]. They used a CRRT platform and, after the hemofilter, a small standard hollow-fiber gas exchanger (D902 Liliput 2 ECMO; Sorin Group Milan, Italy; surface area of 0.67 m^2^). The RRT mode was continuous venovenous hemodialysis (CVVHD). The data showed an average PaCO_2_ reduction of 17.3 mmHg in about 4 h with a concomitant increase in pH. In parallel to the pH correction, a marked stabilization of hemodynamics was observed. At 24 h, the mean TV was reduced from 8.4 to 7.3 mL/kg PBW and the Pplat was reduced from 19.8 to 18.8 cmH_2_O. All the patients tolerated the intervention, and no complications occurred during the therapy. Two episodes of clotting were observed, but no serious adverse events attributed to the hollow-fiber gas exchanger or the CRRT occurred. Seven out of ten patients were successfully weaned from the low-flow CO_2_ removal system, their pulmonary function was improved, and they recovered from critical illnesses. In 2014, Quintard et al. conducted a very similar investigation on 16 patients affected by ARDS treated with CRRT for oliguric AKI [57]. They used a standard device in CVVHD or continuous venovenous hemofiltration (CVVH) modality. An oxygenation membrane, initially designed for pediatric ECMO (HILITE 2400 LT, Medos), was introduced upstream from the hemofilter. The average PaCO_2_ reduction was 24.4 mmHg after 6 h and 30 mmHg after 12 h (31% and 39%, respectively), associated with a pH increase of 0.16 at 6 h and 0.23 at 12 h, respectively. The mean TV was reduced from 5.9 mL/kg PBW before the treatment to 5.5 mL/kg PBW at 12 h. The mean Pplat before the treatments were 27.7 cmH_2_O and 25.6 cmH_2_O at 12 h. No complications or adverse events attributable to the treatment were reported. Seven of the sixteen patients died, but the timing, cause, and place of death were not specified. In 2015, Allardet-Servent et al. conducted a prospective human observational study on eleven patients with ARDS and AKI. CRRT was delivered with a PrismaFlex v6.0 monitor (Gambro, Lund, Sweden) in the CVVH modality and the membrane oxygenator was inserted either upstream or downstream of the hemofilter [58]. On average, the oxygenator blood flow and CO_2_ removal rate were higher when the membrane was put upstream of the hemofilter, but the differences were not statistically significant (PaCO_2_ relative reduction 22 ± 7% upstream vs 18 ± 6% downstream). At the beginning of the treatment, the TV ventilation was fixed at 6 mL/kg PBW, but then it was possible to reduce it to 4 mL/kg PBW. Thereafter, the TV was reduced to 4 mL/kg PBW for the remainder of the study (72 h). However, even in this cohort, the ICU mortality rate remained elevated (nine patients-82%). A point of strength of this study is that, unlike previous investigations, the authors used a standardized protocol of ventilation based on the ARDSNet protocol. In 2018, Fanelli et al. compared thirteen patients treated with ECCO_2_R–CRRT with propensity-score-matched patients treated only with CRRT [59]. They found that after 24h of the combined treatment, it was possible to achieve a significant reduction in TV (from 7.04 ± 0.5 to 4.84 ± 0.4 mL/kg PBW) while maintaining a stable PaCO_2_ level. Interestingly, the authors also observed a significant decrease in inflammation and apoptosis markers in patients undergoing the combined treatment. In 2018 in a multicenter study, Schmidt et al. evaluated twenty patients with mild-to-moderate ARDS, treated with a low-flow CO_2_-removal device, Prismalung^®^ (Baxter Gambro Renal, Deerfield, IL, USA), which consisted of a 0.32m^2^ membrane oxygenator that was integrated into the Prismaflex^®^ platform (Baxter Gambro Renal, USA) [60]. None of the patients had AKI, and so the ECCO_2_R was provided standalone (without concomitant RRT). Additionally, in this case, the ECCO_2_R was helpful in sustaining uLVP and limiting the increase in PaCO_2_. Interestingly, the same ECCO_2_-CRRT configuration was used by Nentwich et al., who, in a multicenter observational study, evaluated twenty hypercapnic patients with concomitant renal failure requiring CRRT [61]. The RRT modality was CVVH, and ventilation parameters were set according to the ARDSNet recommendations. The data showed an average PaCO_2_ reduction of 7.4 mmHg, a concomitant 0.4 increase in pH, and a slight decrease in the VT (from 6.0 ± 0.7 to 5.5 ± 0.8 mL/kg PWB) and Pplat (from 30 ± 4 to 28.9 ± 3.6 cmH_2_O) after 24 h. The combined treatment ameliorated respiratory acidosis and effectively reduced the invasiveness of MV while delivering an efficient renal replacement therapy and reducing the vasopressor requirements. Notably, this study provided the first description of a certified and labeled combination therapy on a commercially available organ support platform. Finally, in 2021, Consales et al. published their retrospective observational study (the CICERO study) carried out between 2016 and 2019 in 22 patients with either mild-to-moderate ARDS or aeCOPD associated with AKI treated with combined ECCO_2_R–CRRT [53]. Similarly to Netwitch et al., they used the PrismaLung^®^-Prismaflex^®^ platform. The average PaCO_2_ was efficiently reduced from 73.8 to 46.6 mmHg in 24 h, and the pH concomitantly increased from 7.20 to 7.40. The treatment allowed 12/17 patients on mechanical ventilation to shift to protective ventilation within 24 h. No complications related to ECCO^2^R–CRRT were recorded. Overall, 21 out of the 22 patients recovered from AKI during their hospitalization, while one patient was on intermittent hemodialysis due to underlying end-stage renal disease before their admission to the ICU. In Figure 1 we present schematic representations of the different configurations of the combined ECCO_2_R–CRRT circuits. Table 1 and Table 2 summarize the main clinical characteristics and operational parameters of the devices used in these studies.

### Experiences with ECCO_2_R Integrated into CRRT Platform in COVID-19 Patients

As already stated, the COVID-19 pandemic has offered the possibility to reevaluate the suitability of ECCO_2_R provided on CRRT platforms. Indeed, the high number of COVID-19 patients suffering from ARDS highlighted the need for a simple and widely available solution to provide the best ventilatory strategy option for these patients, regardless of their renal function [62]. Therefore, unlike previous experience, during the pandemic, ECCO_2_R–CRRT combined treatment was mostly used in patients without AKI or renal failure. Moving beyond single-case reports [63], in 2020, Husain-Syed et al. treated four COVID-19 patients complicated by ARDS with an ECCO_2_R (multiECCO_2_R, Eurosets) in conjunction with multiFiltrate CRRT platforms (Fresenius Medical Care, Bad Homburg, Germany) [64]. Three patients received ECCO_2_R standalone with the multiFiltrate set in the hemoperfusion mode, and one patient, who suffered from AKI, received ECCO_2_R coupled with CRRT in the CVVHD mode. The multiECCO_2_R was inserted in a series after the hemofilter (Ultraflux AV 1000S, Fresenius Medical Care, Bad Homburg, Germany). The ECCO_2_R–CRRT was commenced at a blood flow of 200 mL/min. Regional citrate anticoagulation plus systemic heparinization were used as an anticoagulation strategy. In two patients, it was necessary to implement the blood flow rate to 400 mL/min to achieve good PaCO_2_ clearance. The average PaCO_2_ reduction was 15.4 mmHg after 24 h of treatment, and the pH increased from 7.33 ± 0.07 to 7.45 ± 0.07. After 24 h, it was possible to decrease the TV and Pplat. No ECCO_2_R–CRRT-related adverse events occurred. The ECCO_2_R treatment was terminated after a median of 5.5 (4.5–7.5) days due to a sustained improvement in hypercapnia. In the AKI patient, CRRT was continued for another four days because of oliguria. During the first wave of the pandemic, Ding et al. conducted a single-center study on 12 patients affected by COVID-19 ARDS with refractory hypercapnia (PaCO_2_ > 50 mmHg) admitted in the ICU of Wuhan [65]. They used a low-flow gas-exchanger oxygenator integrated into the Prismaflex platform (Gambro-Baxter) to decrease the PaCO_2_ level and permit a low Pplat and driving pressure ventilation. In this case, the patients did not suffer from AKI, so the CRRT machine was set in the slow continuous ultrafiltration (SCUF) mode with an ultrafiltration rate of 0 mL. The mean blood flow was 342.5 ± 49.20 mL/min, and the CO_2_ clearance reached the best efficiency (45.91 ± 7.70 mL/min) at a sweep gas flow of 10 L/min. After the application of the ECCO_2_R device, the PaCO_2_ in all the patients decreased. The treatment led to an 8.48 cmH_2_O reduction in the Pplat in 24 h. Even in this cohort, the combined ECCO_2_R–CRRT treatment was safe, and no major adverse events were reported. Nevertheless, the 28-day mortality was high (67%). Finally, in 2022, Alessandri et al. retrospectively reported their experience in the treatment of 27 patients with ARDS and AKI requiring invasive mechanical ventilation undergoing ECCO_2_R–CRRT [66]. The initiation of the treatment reduced the TV from 6.0 ± 0.6 mL/kg to 4.3 ± 0.3 mL/ mL/kg PWB and the Pplat from 28.9 ± 2.7 to 21.6 ± 2.8 cmH_2_O with a reduction in the respiratory rate. Throughout the course of the ECCO_2_R, these changes were accompanied by the stabilization of PaCO_2_ and an increase in pH. Simultaneously, the combined treatment was associated with a significant reduction in the serum creatinine levels. No major adverse effects occurred, but 17 patients (63%) died within 28 days. Table 3 and Table 4 summarize the main clinical characteristics and operational parameters of the devices used in COVID-19 patients with ARDS.

## 7. Critical Considerations 

Here, we have reviewed the principal available experiences and evidence of combined ECCO_2_R–CRRT in various clinical settings, such as ARDS of different etiologies, including COVID-19, and aeCOPD in patients with or without associated renal failure. The critical analysis of these data allows us to make some generalizations. As a point of strength, all the studies agree in that they suggest that ECCO_2_ R alone or set on a CRRT platform effectively controls hypercapnia and respiratory acidosis in MV patients. This is a crucial issue because the regulation of PaCO_2_ levels is essential to permitting the adoption of LPV strategies [67]. Furthermore, during ECCO_2_R–CRRT, CO_2_ removal may be obtained using low blood flow, thus facilitating clinical management and reducing treatment-related adverse effects. On the other hand, the reported findings present many weaknesses. The most relevant limitation is that, currently, there is no evidence of the effects of ECCO_2_R and ECCO_2_R–CRRT in improving patient outcomes and reducing mortality [68]. This is a result often found in studies investigating critically ill patients, which may be a consequence of the clinical complexity of these patients but also of the small sample size and short time of treatment that characterizes these studies [69]. Moreover, they present a high heterogeneity since different patient populations, outcomes, devices, and operative parameters were investigated. Similarly, with some exceptions, there is a lack of a standardized ventilation protocol without prefixed objectives. These aspects significantly reduce the generalizability of the reported data. Furthermore, it should be noted that in studies involving patients with renal failure, the renal outcome and recovery were been poorly reported; thus, the adequacy of renal support provided by ECCO_2_R–CRRT systems is unclear. Finally, many other issues have not been sufficiently explored. For example, we need to investigate the most efficient circuit configuration (i.e., the position of the membrane oxygenator and hemofilter may impact the circuit performance), the effects of a dialysis buffer on the systemic acid–base balance, and the management of anticoagulation with the possible use of citrate. All these considerations underline the need for further studies and suggest caution in translating experimental evidence into clinical practice.

## 8. Conclusions

Extracorporeal CO_2_ removal techniques offer several advantages for ventilatory strategy optimization in patients with respiratory failure. However, although the different studies demonstrated the efficacy of ECCO_2_R in improving hypercapnia and metabolic acidosis, this treatment is not risk-free, and its impact on the prognosis of critically ill patients is undefined. Notably, these patients are characterized by high complexity, with multiorgan involvement, often requiring a multidisciplinary approach [70]. In this sense, the combination of different extracorporeal support techniques could offer clinical benefits in terms of the reduction in complications, as well as organizational and economic advantages. The possibility of using ECCO_2_R coupled with CRRT platforms provides an example of this approach. First, the ECCO_2_R–CRRT combination is flexible since it can be employed in patients with respiratory failure, including those with COVID-19, with or without concomitant renal disease. Furthermore, exploiting widely available equipment, such as those required for CRRT, it can also be used in non-highly specialized centers, as it does not require specific training. For the same reason, ECCO_2_R–CRRT could save time and costs compared to equipment specifically designed for ECCO_2_R. The disadvantage is that the ECCO_2_R circuit integrated into the CRRT only allows for low-flow techniques, which may be insufficient for some patients. On the other hand, the low-flux treatment with ECCO_2_R–CRRT seems to be well tolerated and not burdened by significant adverse events, except for the risk of circuit coagulation. Nevertheless, as discussed above, the available evidence presents many limitations, so an ideal approach would be to wait for specifically designed randomized clinical trials to determine the actual clinical impact of ECCO_2_R and ECCO_2_R–CRRT. However, admittedly, in critically ill patients, large clinical trials are not easy to implement. So, the active reporting of clinical experiences and cohort studies is essential to defining and confirming the suitability and safety of this approach as well as identifying patients who can benefit the most from this therapy.

## Figures and Tables

**Table 1:** Design, patient characteristics, and outcome of studies reporting use of combined ECCO_2_R–CRRT treatment.

Study, Ref	Study Design	Patients, n	Patient Characteristics (%)	Patients with Renal Failure, n (%)	PaCO_2_ (mmHg)/ pH Baseline	PaCO_2_ (mmHg)/ pH End	Main Outcomes	AE
Terragni 2009 [39]	Prospective cohort study	32	Pneumonia (34) Sepsis (50) Trauma (16)	0	73.6 ± 11 7.2 ± 0.02	47.2 ± 8.6 7.38 ± 0.04	Reduction in TV in patients with initial high Pplat	Membrane clotting in three pts
Forster 2013 [56]	Pilot study	10	H1N1 pneumonia (30) Bacterial pneumonia (50) aeCOPD (20)	AKI: 10 (100)	69 ± 10.5 7.18 ± 0.8	53.6 ± 13.5 7.29 ± 0.07	Seven pts weaning from MV Two pts died in ICU	System clotting in two pts
Quintard 2014 [57]	Retrospective single-center study	16	ARDS with Pneumonia (56) Shock (19) Other (25)	AKI: 16 (100)	77.4 ± 13.4 7.17 ± 0.1	47.4 ± 9.7 7.40 ± 0.07	Reduction in TV Seven pts (43%) died in ICU	None
Allardet-Servent 2015 [58]	Prospective observational study	11	ARDS with Pneumonia (27) Urinary infection (36) Peritonitis (18) Other (18)	AKI: 11 (100)	47 ± 11 7.28 ± 0.12	37 ± 4 7.42 ± 4.8	PaCO_2_ reduction during LPV High mortality in ICU (82%)	Hemofilter clotting in one pt
Fanelli 2018 [59]	Prospective cohort study—propensity score matching	13 ECCO_2_R–CRRT Vs 13 CRRT standalone	ARDS, not specified	AKI: 26 (100)	NA	NA (reported as stable)	In ECCO_2_R–CRRT group: uLPV reduced inflammatory and apoptosis marker	None
Schmidt 2018 [60]	Prospective observational study	20	Mild/Moderate ARDS Pneumonia (80) Other (20)	0	43 ± 8 7.39 ± 0.1	53 ± 9 7.32 ± 0.1	Limited PaCO_2_ increase during LPV 28-day mortality 15%.	Membrane clotting in ten pts Two cases hemoptysis
Nentwich 2019 [61]	Multicenter observational pilot study	20	ARDS (65) arCOPD (35)	AKI: 14 (70) CIHD: 6 (30)	68.3 ± 11.8 7.18 ± 0.09	53.2 ± 14.7 7.22 ± 0.08	Improvement of ventilatory parameters and reduction in norepinephrine	Circuit clotting in five pts
Consales 2021 [53]	Retrospective single-center observational study	22	ARDS (36) aeCOPD (64)	AKI: 18 (82) CKD: 4 (18)	73.8 ± 11.3 7.20 ± 0.02	43.5 ± 4 7.40 ± 0.02	Shift to LPV in 62% of MV pts 21 pts recover from AKI Mortality 27%	None

Data are expressed as Mean ± SD. Abbreviations: ECCO_2_R = Extracorporeal CO_2_ Removal; CRRT = Continuous Renal Replacement Therapy; ARDS = Acute Respiratory Distress Syndrome; aeCOPD = acute exacerbation of Chronic Obstructive Pulmonary Disease; AKI = Acute Kidney Injury; CIHD = Chronic Intermittent Haemodialysis; CKD = Chronic Kidney Failure; TV = Tidal Volume; MV = Mechanical Ventilation; LPV = lung-protective ventilation; ICU = Intensive Unit Care; pts= patients; NA = data not available; AE = adverse effects.

**Table 2:** Devices and operative parameters of studies reporting use of combined ECCO_2_R–CRRT treatment.

Study, Ref	ECCO_2_R Device	CRRT Platform	CRRT Modality	ECCO_2_R Position *	Circuit Duration (h)	Anticoagulant	Blood Flow (mL/min)	Membrane Oxigenator Area (m^2^)	Sweep Gas Flow (L/min)	CO_2_ Removal (mL/min)
Terragni, 2009 [39]	Decap^®^, Hemodec	Hemofilter MedicaD200, Medolla, Ita	NA	Pre	144 (84–168)	Heparin	191–422	0.33	8	NA
Forster 2013 [56]	D902 Liliput 2 ECMO; Sorin Group	bm11/14; Edwards-Lifescience, Irvine	CVVHD	Post	24	Heparin	378 ± 85.3	0.67	5.2 ± 0.98	NA
Quintard 2014, [57]	HILITE 2400 LT, MEDOS	Multifiltrate, Fresenius MedicalCare	CVVHD/ CVVH	Pre	5.9 ± 3.8 days	Heparin	400–500	0.65	10	NA
Allardet-Servent 2015 [58]	HILITE 2400 LT, MEDOS	PrismaFlex v6.0 monitor Baxter Gambro	CVVHF	Pre: 7 pts Post: 5 pts	72	Heparin	Pre: 432 ± 25 Post: 382 ± 29	0.65	8	Pre: 91 ± 49 Post: 72 ± 59
Fanelli 2018 [59]	NA	Diapact; B. Braun Avitum	NA	Pre	NA	Heparin 6 pts Citrate 7 pts	276 ± 53	NA	8.1 ± 0.5	NA
Schmidt 2018 [60]	Prismalung™, Baxter Gambro	PrismaFlex v6.0 Baxter Gambro	Not applied	NA	31 ± 22	Heparin	421 ± 40	0.32	10 ± 0.3	51 ± 26
Nentwich 2019 [61]	Prismalung™, Baxter Gambro	PrismaFlex v6.0 Baxter Gambro	CVVHF	Post	95.8 ± 47.7	Heparin	400–500	0.32	NA	43.4 ± 14.1
Consales 2021 [53]	Prismalung™, Baxter Gambro	PrismaFlex v6.0 Baxter Gambro	CVVHDF	NA	82.9 ± 31.2	Heparin	217 ± 88.2	0.32	6.4 ± 4.9	NA

Data are expressed as Mean ± SD or Median (ranges). Abbreviations: ECCO_2_R = Extracorporeal CO_2_ Removal; CRRT = Continuous Renal Replacement Therapy; CVVHD = Continuous Venovenous Haemodialysis; CVVH = Continuous Venovenous Haemofiltration; CVVHF = Continuous Venovenous Haemofiltration; pts = patients; NA = data not available; AE = adverse effects. * ECCO_2_R position is indicated as Pre or Post for when membrane oxygenator is placed upstream or downstream of the hemofilter, respectively.

**Table 3:** Design, patient characteristics, and outcome of studies reporting use of combined ECCO_2_R–CRRT treatment in COVID-19 patients with ARDS.

Study, Ref	Study Design	Patients, n	Patients with Renal Failure, n (%)	PaCO_2_ (mmHg)/ pH Baseline	PaCO_2_ (mmHg)/ pH End	Main Outcomes	AE (%)
Husain-Syed 2020 [64]	Single-center, prospective	4	1 (25)	60.7 ± 5.4 7.33 ± 0.07	47 ± 3.7 7.42 ± 0.05	TV and Pplat reduction, no effect on hemodynamics	None
Ding 2021 [65]	Single-center, prospective	12	0	64.5 (56–88.75) 7.33 (7.22–7.41)	66.4 (44.3–95.9) NA	TV and Pplat reduction, 28-day mortality 67%	None
Alessandri 2022 [66]	Multicenter retrospective study	27	AKI: 27 (100)	68.1 ± 11.2 7.30 ± 0.08	NA (stable) 7.39 ± 0.08	TV reduction. Renal function improvement 28-day mortality 63%.	Circuit clotting in four pts

Data are expressed as Mean ± SD or Median (ranges). Abbreviations: ECCO_2_R = Extracorporeal CO_2_ Removal; CRRT = Continuous Renal Replacement Therapy; ARDS = Acute Respiratory Distress Syndrome; AKI = Acute Kidney Injury; TV = Tidal Volume; Pplat = Plateau Pressure; pts= patients; NA = data not available; AE = adverse effects.

**Table 4:** Devices and operative parameters of studies reporting use of combined ECCO_2_R–CRRT treatment in COVID-19 patients with ARDS.

Study, Ref	ECCO_2_R Device	CRRT Platform	CRRT Modality	ECCO_2_R Position *	Circuit Duration (h)	Anticoagulant	Blood Flow (mL/min)	Membrane Oxigenator Area (m^2^)	Sweep Gas Flow (L/min)	CO_2_ Removal (mL/min)
Husain-Syed 2020 [64]	MultiECCO2R; Eurosets	Multifiltrate, Fresenius Medical Care	Hemoperfusion (3 pts) CVVHD (1 pt)	Post	5.5 days	Heparin + Regional Citrate	350 ± 87	1.35	5.4 ± 1	NA
Ding 2021 [65]	QUADROX-I pediatric HMO30000, MAQUET	Prismaflex platform, Gambro-Baxter	SCUF (with UF = 0)	Pre	24 h	Heparin	342.5 ± 49	0.8	10	45.91 ± 7.70
Alessandri 2022 [66]	OMNI blood purification System, B.Braun Avitum	OMNI blood purification system	CVVHDF (15 pts) CVVHD (6 pts) CVVH (6 pts)	Pre	>48 h	Heparin	186–393	1.81	9–11	NA

Data are expressed as Mean ± SD. Abbreviations: ECCO_2_R = Extracorporeal CO_2_ Removal; CRRT = Continuous Renal Replacement Therapy; CVVHD = Continuous Venovenous Haemodialysis; SCUF = Slow Continuous Ultrafiltration; UF = ultrafiltration; CVVH = Continuous Venovenous Haemofiltration; pts = patients; NA = data not available; AE = adverse effects. * ECCO_2_R position is indicated as Pre or Post for when membrane oxygenator is placed upstream or downstream of the hemofilter, respectively.

## Data Availability

Not applicable.
